# The impact of interaction with children on internet addiction in older adults: A moderated mediation model

**DOI:** 10.3389/fpsyg.2022.989942

**Published:** 2022-08-23

**Authors:** Yang Yang, Tianyuan Liu, Yu Jia

**Affiliations:** ^1^School of Sociology, Wuhan University, Wuhan, China; ^2^School of Journalism and Communication, Wuhan University, Wuhan, China

**Keywords:** older adults, internet addiction, loneliness, life satisfaction, interaction with children

## Abstract

Internet addiction among older adults is a new problem in many countries. However, previous studies on excessive Internet use have focused more on young people, and only few studies have focused on Internet addiction in older adults. There is a need to continue to expand research on Internet addiction in older adults. This paper aimed to fill the gap in exiting literature. We adopted a self-reported questionnaire to assess the elderly’s interaction with children, loneliness, life satisfaction and Internet addiction among old adults. A total of 241 old people were obtained from data collection in China *via* online survey with the help of a professional research company. We used OLS regression analysis and bootstrap method to test the hypothesis. The results of the empirical analysis indicated that (1) interaction with children was significantly negatively associated with the Internet addiction of old people; (2) loneliness mediated the relationship between interaction with children and old adults’ Internet addiction; and (3) life satisfaction moderated the effect of interaction with children, and the indirect effect between interaction with children and old adults’ addiction *via* loneliness was stronger for those with low life satisfaction. Finally, we discussed the theoretical significance, practical implications, limitation of this research. Interventions to improve family function systems especially for older people with low life satisfaction can help prevent the development of Internet addiction.

## Introduction

Over the past two decades, the Internet has been widely used across all age groups in such a digital world. The 49th Statistical Report on the Development Status of the Internet in China released by the China Internet Network Information Center notes that as of December 2021, the size of elderly Internet users aged 60 and above have reached 119 million, and the Internet penetration rate have reached 43.2%. As we can see, it is an increasingly common trend for the elderly to be online. However, Internet addiction in older adults is an emerging area, as previous literature has focused more exclusively on the benefits of Internet use in this age group (Silva et al., 2022; Yuan et al., 2022). Compared to the youth, the older people have worse health situation and are prone to use Internet excessively due to factors such as physical function and need to supplement social resources through the Internet (Brailovskaia et al., 2021).

In fact, Internet addiction among the elderly has begun to receive social attention and exists to a certain extent. For example, China’s Internet Life of the Elderly 2020 report shows that 0.19% of seniors are online for more than 10 h a day on some apps, and more than 100,000 seniors live on the mobile web almost 24/7. The average daily usage time of senior users over 60 years old reached 64.8 min and each senior user logged into the app five times a day on average. Half of the old experience states such as inability to adapt or anxiety when cell phones are not accessible to the Internet, which are typical of Internet addiction withdrawal reactions (Zhu et al., 2018). Meanwhile, researchers have examined that higher problematic media use was associated with high perceived social isolation among the elderly (Meshi et al., 2020). These reports and studies show that Internet addiction is not only a problem for young people but is gradually becoming a problem that older people may encounter as well. During the COVID-19 pandemic, more and more older adults are using the Internet for long periods of time to connect society with others and get news, which may increase their risk of addiction. Compared to the youth, older people have a shorter exposure to the Internet and various smart devices. Therefore, the novelty brought to them by the Internet is much higher than that of the younger generation and it is also more likely to produce addiction problems (Wang, 2021). However, previous research has established that addiction to social media can damage individuals’ mental health, indicating depression, anxiety, loneliness, psychological distress, sleep disorders, and eating disorders (Kuss and Griffiths, 2011; Keles et al., 2020; Cunningham et al., 2021; Lane et al., 2021; Huang, 2022). And in some cases, problematic social media use may lead to clinical treatment and some physical pain (Karaiskos et al., 2010; Griffiths et al., 2014; Zheng et al., 2016). For older adults, the damage of addiction can be even more severe. Because they are approaching the later stages of life and are gradually deteriorating in all aspects of physical functioning. Considering these, more attention should be paid to Internet addiction of the elderly, because of the negative health consequences caused by excessive Internet use (Jiang and Song, 2022). Additionally, the mechanisms of Internet addiction in older adults should also be explored to find ways to reduce their addiction.

However, as far as we know, little attention was paid to the negative effects of excessive internet usage of the old. Only little research paid attention to the factors that influence Internet addiction among the elderly. For example, depressive symptoms, social support from family and from a significant other had significant effects on the older adults’ addictive use of social media (Özbek and Karaş, 2022). According to social support theory, the psychological support and material resources provided by social networks to individuals can help them cope effectively with the stressful challenges in their lives (Cohen, 2004). As a result, perceived social support can significantly reduce online addiction (Wu et al., 2016). In particular, offline social support protects mental health and reduces the likelihood of Internet addiction (Meshi and Ellithorpe, 2021). Compared to some western countries, China is a country where family intergenerational relations are of prominence. Hence, family is a very important source of support for Chinese, especially for the old. Elderly who enjoyed a positive intergenerational relationship with their children would experience adequate family social support (Li et al., 2019). Although some studies have focused on the relationship between family support and Internet addiction in older adults (Özbek and Karaş, 2022). Nevertheless, they did not focus on the important impact of emotional support from family on Internet addiction in older adults. Financial support and caregiving support require high financial resources as well as time from the children themselves, while emotional support is the support resource that can most efficiently enhance the family support system in a short period of time from a practical point of view. And emotional support has been proven to be more effective for the mental health of older adults (Rekawati et al., 2019). Therefore, it is necessary to explore the relationship between emotional support and Internet addiction among older adults. Increasing interaction with children is the simplest and most practical operation to enhance emotional support. However, no articles have focused on the relationship between the interactions with children and Internet addiction among older adults.

In addition, some studies have also focused on the mediating mechanisms of the effect of the level of social participation on Internet addiction among older adults. It has been noted that social participation is indirectly related to two dimensions of Internet addiction, *via* the social influence that promotes the use of technology, which has implications in development of interventions that encourage Internet use in older persons (Rosell and Vergés, 2020). But no studies have focused on the mediating mechanisms between interactions with children and Internet addiction in older adults. The psychological distress model suggested that loneliness are psychological variables that are very relevant to Internet addiction (Kandell, 1998). No attention was paid to the relationships and mechanisms between interaction with children, loneliness, and Internet addiction of the old. Besides, previous studies on the antecedents of Internet addiction in older adults have focused more on which factors are associated with Internet addiction in older adults but neglected the role boundaries and applicable conditions of their relationship (Meshi et al., 2020; Rosell and Vergés, 2020; Özbek and Karaş, 2022). Therefore, we aimed to investigate what factors moderate the role of factors influencing Internet addiction in older adults. Life satisfaction can help explain why some are more prone to Internet addiction while others are less, with the explanation that greater life satisfaction was associated with decreased Internet addiction (Lachmann et al., 2018). Whether the mechanism linking interactions with children with Internet addiction depends on life satisfaction was still unknown for elderly people.

**Figure 1:**
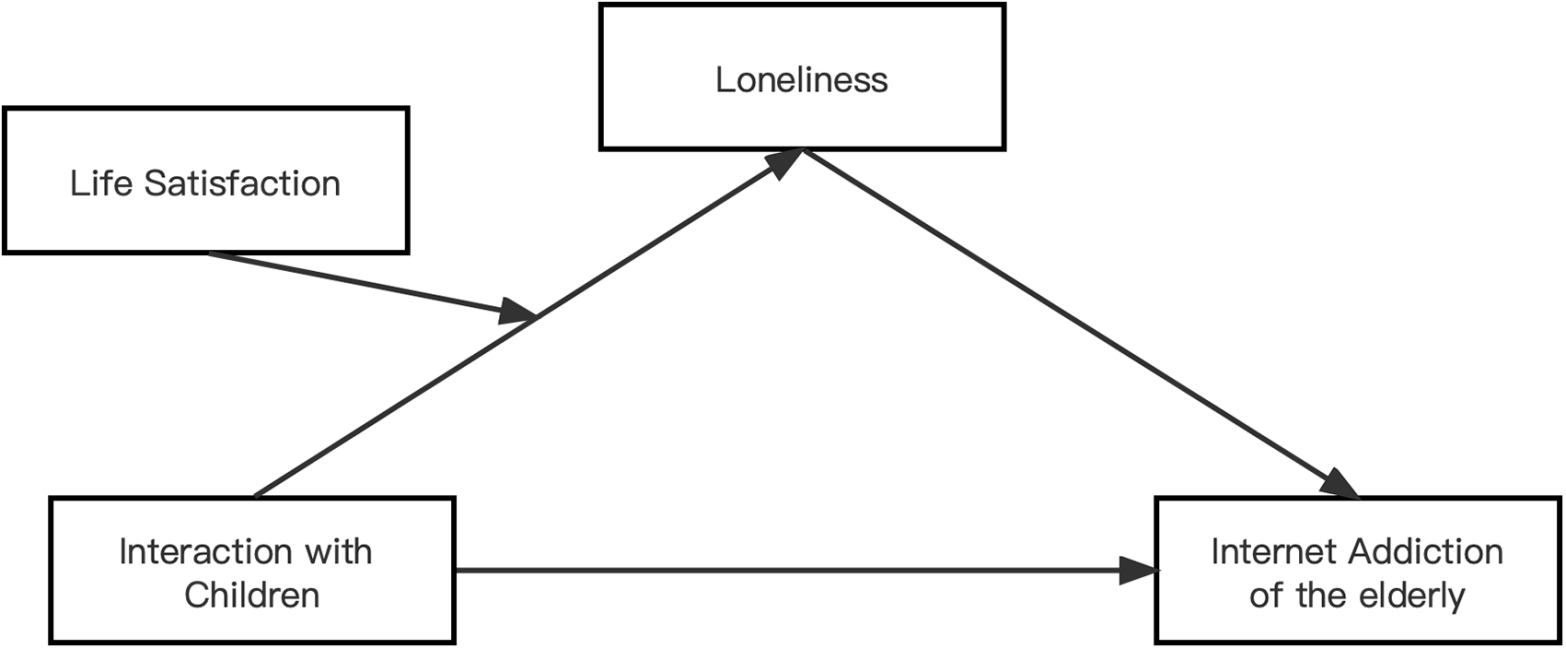
The conceptual model of this study.

In summary, this study aims to fill the gap in the existing literature about Internet addiction among the elderly by presenting a case study of mainland China. First, based on social support theory, we analyze the impact of interaction with children on Internet addiction among the elderly. Second, we examine how interaction with children affect Internet addiction among older adults. Specially, loneliness plays a mediating role between interaction with children and Internet addiction among the old. Finally, by testing the moderating mechanism of life satisfaction in aforementioned relationships, we reveal the boundary condition of the effect of interaction with children on Internet addiction among the elderly. Figure 1 is the conceptual model.

## Literature review and hypothesis

### The relation between interaction with children and internet addiction

Social support is considered to be general or specific supportive behaviors from others that enhance an individual’s social adjustment and protect the individual from adverse circumstances (Demaray and Malecki, 2002). Social support plays a role in both links of the chain of relationships between stressful events and health status (Cohen and Wills, 1985). However, once the desired social support is not available in real life, some individuals tend to obtain social support or meet their needs through the Internet (Yao and Zhong, 2014). Nevertheless, long-term Internet use also harbors the risk of addiction, and Internet addiction is a challenge that older adults need to address in their Internet use because of the negative health consequences caused by excessive Internet use (Jiang and Song, 2022). The elderly are more likely to have mobility impairments and unable to connect with their social relationships offline, loneliness may be exacerbated if they use the internet excessively (Meshi et al., 2020). How to reduce or even eliminate Internet addiction was gradually gaining attention. Numerous studies have shown that social support negatively predicts Internet addiction (Wang and Zhang, 2020) and social relationships have an important impact on social support (Leimeister et al., 2008), which improves individual adaptability by increasing positive emotions, self-confidence, and self-management (Heller et al., 1986). That is, increased social support enhances individuals’ resilience and therefore enables more rational and healthy use of the Internet, further reducing the risk of Internet addiction.

Social interaction provides empathic support for individuals (Gee et al., 2006). Traditional friendships based on face-to-face interaction are generally considered to provide social support, social identity, and a sense of belonging (Hamm and Faircloth, 2005). Importantly, family factors were pointed out as an important factor in Internet addiction. A study indicated that parent-adolescent interactive communication was significantly related to adolescent Internet addiction (Xu et al., 2014). Similarly, Chinese seniors have important interdependent intergenerational relationships with their children. The Chinese emphasize collectivism, and the family is the main source of social support in Chinese culture, so intergenerational relationships play an important role throughout the life cycle (Li and Dong, 2020). Better intergenerational relationships between older adults and their children mean more family support (Li et al., 2019). Despite the financial support, emotional support is another major benefit of a family support system, which is of crucial importance for the elderly when facing problems and challenges to improve their health and well-being (Rekawati et al., 2019). Interaction with children is a form of support and offline social interactions can negatively affect Internet addiction (Yao and Zhong, 2014). The help of the younger generation can increase the interest and skills of the elders in using the Internet, which is a “bottom-up technology transfer” (Correa, 2014; Eynon and Helsper, 2015). Thus, during the interaction, children can teach their parents how to use the Internet properly, which can reduce the old people’s risk of Internet addiction. Consequently, we speculate that when older adults have more interactions with their children, more family support is supplied to older adults, which will reduce their risk of Internet addiction.

Therefore, we propose the following hypothesis:

*H1*: Interaction with children can negatively affect older adults' Internet addiction.

### The mediating role of loneliness

Loneliness can be defined as a subjective experience of social isolation and is typically characterized by feelings of social disconnection (Lim and Gleeson, 2014). Loneliness is a serious problem for older adults and can be alleviated through social support. Aging is accompanied by a decline in physical and cognitive functioning and a loss of social network relationships and resources, which increases older adults’ need for social support and vulnerability to social stress, which may trigger or contribute to feelings of loneliness (Chen (Yixin) and Feeley, 2014). Loneliness, however, may vary with the number of social contacts, defined as an individual’s daily social interactions (Jones, 1981), that one has (Gross et al., 2002). Studies have found that low frequency of social contact with family and friends is associated with loneliness in older adults (Bondevik and Skogstad, 1998). For older adults, a lack of close social contact is more likely to induce loneliness than a lack of regular social contact (Green et al., 2001). This close social contact is an important source of social support in later life. Interactions with children are an important manifestation of intimate social contact and an important source of emotional support for older adults. Fortunately, high levels of social connectedness are associated with low levels of loneliness (Grover et al., 2018).

On the other hand, the psychosocial distress model argues that one of the important motivations that drive individuals to become addicted to the Internet is to use the Internet to cope with and alleviate individuals’ existing psychological problems (Kandell, 1998). Thus, lonely people are particularly vulnerable to Internet addiction, with lacking social interaction and social support in real life (Bozoglan et al., 2013). It is possible to say that lonely individuals with poor social skills have difficulties to manage healthy social relationships. As result of this, they would be prone to experience problematic internet use and psychological problems, due to the isolation from the real life. Overall, based on the above-mentioned, we propose the following hypothesis:

*H2*: Loneliness mediates the negative relationship between interaction with children and Internet addiction in old adults.

### The moderating role of life satisfaction

Life satisfaction is an important psychological force, even a “core construct in positive psychology” (Proctor et al., 2009). Previous studies have indicated that life satisfaction is a potentially important protective factor against adverse or stressful life events (Chai et al., 2018). Based on the theory of stress and coping, life satisfaction is considered to be a general evaluation style that will influence people’s immediate cognitive evaluations of specific environmental events, which theoretically explains the protective effect of life satisfaction (Lazarus, 1993). In this case, people with higher life satisfaction will evaluate stressful situations in a more positive way and develop more positive coping strategies, which can weaken the negative impact of stressful events on individuals. Conversely, people with lower life satisfaction will develop coping strategies in a more negative manner, which can enhance the positive influence of advantageous resources on individuals. In short, life satisfaction as a positive psychological force can break down or squeeze the positive effect of other positive resources in coping with stressful situations or events.

Additionally, life satisfaction is regarded as a protector from loneliness (Andrew and Meeks, 2018). Older adults often experience reduced social support, isolation, and low levels of life satisfaction due to the loss of social interactions (Shillair et al., 2015). Social support improves individual adaptability by increasing positive emotions, self-confidence, and self-management (Heller et al., 1986) and interaction with children is a form of social support. What’s more, it has been demonstrated that life satisfaction is a vital variable to enhance several indicators of psychosocial health, such as loneliness, in older adults (Kim et al., 2021). Hence, to test the role of life satisfaction as a psychological force, further research is needed to investigate whether life satisfaction can moderate the link between advantageous resources (i.e., interaction with children) and the development of psychological problems (i.e., loneliness) in older adults.

Therefore, we can assume that older adults with low life satisfaction have more difficulties maintaining good relationships and are more likely to experience loneliness. Consequently, an increase in interaction with children can more significantly reduce loneliness. Thus, the following hypothesis is proposed:

*H3*: Life satisfaction moderates the relationship between interaction with children and loneliness in old adults, such that the negative relationship is stronger when the older adults possess lower life satisfaction.

Also, on the basis of the previously discussed assumptions that when considering the old, interaction with children can negatively affect Internet addiction, and loneliness is positively associated with internet addiction, and the negative effect of interaction with children is stronger on loneliness when the elderly possesses lower life satisfaction, it is logical to speculate that the negative indirect impact of interaction with children on Internet addiction through loneliness would be stronger for those older adults who have lower life satisfaction. Consequently, the hypothesis is:

*H4*: Life satisfaction moderates the indirect effect of interaction with children on older adults’ Internet addiction via loneliness. Specifically, the indirect effect will be strengthened for those older adults who have lower life satisfaction.

## Materials and methods

### Procedure

We recruited participants aged 55 years and they *via* online survey with the help of a professional research company, which built a large database containing a group of aging network users. The data company sent our survey to its members randomly. Participants were incentivized to complete our survey by five yuan per person. This fee does not include payments to the research company, but is only an incentive for the subjects.

### Participants

The final sample size consisted of 241 participants, after excluding 5 survey respondents who were younger than 55 years old, two survey respondents who did not have children and 13 survey respondents who failed to pass the screening questions. For descriptive statistics about sample demographics, please see Table 1. Of the 241 usable samples, there were 118 female and 123 male participants, ranging in age from 56 to 84 years (*M* = 67.89, SD = 5.90). With regards to education level, the proportions of those with primary degrees or below, those with secondary degrees, those with high school degrees, those with associate degrees, and those with bachelor’s degrees or above were 39.8, 20.3, 23.2, 9.1, and 7.5%, respectively. The number of their children is ranging from 1 to 7 (*M* = 2.40, SD = 1.25), only 30.3% lived with their children and the rest did not. But there was 42.3% need to take care of grandchildren for their own children. Also, the average daily time elderly people spent on internet was 2.54 h (SD = 1.72).

**Table 1:** Descriptive statistics and correlations among all variables.

Variables	(1)	(2)	(3)	(4)	(5)	(6)	(7)	(8)	(9)	(10)	(11)
(1) Internet addiction	1										
(2) Interaction with children	−0.145^*^	1									
(3) Loneliness	0.209^**^	−0.231^***^	1								
(4) Life satisfaction	−0.145^*^	0.139^*^	−0.528^***^	1							
(5) Age	−0.207^**^	0.126	−0.126	0.086	1						
(6) Gender	−0.040	−0.139^*^	0.017	−0.032	0.005	1					
(7) Education level	0.128^*^	0.016	−0.099	0.059	−0.126	0.186^**^	1				
(8) Number of children	−0.232^***^	0.083	0.031	0.046	0.304^***^	0.063	−0.303^***^	1			
(9) Living with children	0.127^*^	0.326^***^	0.036	−0.120	−0.041	−0.185^**^	−0.047	−0.119	1		
(10) Taking care of grandchildren.	0.225^***^	0.253^***^	0.088	−0.013	−0.253^***^	−0.035	0.129^*^	−0.142^*^	0.331^***^	1	
(11) Estimated daily time on Internet	0.550^***^	−0.003	0.031	−0.132^*^	−0.087	−0.010	0.123	−0.128^*^	0.216^**^	0.091	1
Mean	2.078	3.967	2.096	3.471	67.888	0.510	2.241	2.403	0.303	Mean	2.078
SD	0.700	1.012	0.597	0.724	5.901	0.501	1.272	1.248	0.460	SD	0.700

* *p* < 0.05;

** *p* < 0.01;

*** *p* < 0.001.

*N* = 241.

Gender: 1—male, 0—female. Education level: 1—primary degrees or below, 2—secondary degrees, 3—high school degrees, 4—associate degrees, 5—bachelor’s degrees or above. Ling with children: 1—yes, 0—no. Taking care of grandchildren: 1—yes, 0—no.

### Measurement

#### Main variables

We asked the participants questions regarding their Internet addiction, interaction with children, loneliness, and life satisfaction:

##### Internet addiction of the elderly

Internet addiction was measured by the revision of Chinese Internet Addiction Scale for Older Adults, which was revised by us based on the work of Chen et al. (2003). It was employed to assess older adults’ internet addiction on a 27-item self-report instrument on a five-point Likert scale ranging from 1 (strongly disagree) to 5 (strongly agree), with higher scores representing a higher internet addiction level. For the current study, Cronbach’s *α* was high with a Cronbach value of.95 for the entire scale.

##### Interaction with children

Referring to the study by Cao et al. (2014) and Hubatková and Kafková (2017), we assessed participants’ interaction with children with one survey item. We asked, “In general, how often do you interact with your children?” and provided participants with the following five response options: Very infrequent; Relatively infrequent; Fairly; More frequent; Very frequent. Thus, higher scores representing a higher level of interaction with children.

##### Loneliness

We used ULS-8 Loneliness Scale (Hays and DiMatteo, 1987; Zhou et al., 2012) to assess participants’ loneliness. In the present study, the ULS-8 loneliness scale consists of 6 “lonely” positive-order items and 2 “non-lonely” negative-order items, each of which is scored on a five-point frequency scale of 1(never), 2 (rarely), 3 (sometimes), 4 (often) and 5 (always), with positive statements (i.e., non-lonely items) scored in reverse order. The higher the scores were, the greater the loneliness was. Reliability estimates for the total scale obtained in the present study was high, with a Cronbach value of.85.

##### Life satisfaction

We used the Satisfaction with Life Scale (SWLS) to assess participants’ satisfaction with life (Diener et al., 1985; Corrigan et al., 2013). The scale is a 5-item scale designed to measure overall cognitive judgments of one’s life satisfaction. And in the present research, participants indicate how much the agree or disagree with each of the 5 items using a 5-point scale that ranges from 5 strongly agree to 1 strongly disagree. Obtaining high scores on this measure indicated a high level of life satisfaction. The Cronbach’s reliability estimate was acceptable, with Cronbach’s *α* was.82 for the entire scale.

#### Control variables

To eliminate potential confounding effects, referring to the study by Meshi et al. (2020) and Özbek and Karaş (2022), we took demographic information of the employees, including age, gender, education level, living with children, and estimated daily time on internet, as control variables. Meanwhile, we also controlled for number of children and taking care of grandchildren. The reason why these two variables are controlled is based on the following considerations. The greater the number of children, the greater the number of people the older person can interact with. Caring for grandchildren may facilitate ongoing positive relationships with their children and grandchildren (Quirke et al., 2019), which means there may be more interaction between older adults and their children. Both variables directly affect the frequency of interaction between older adults and their children.

##### Age

We asked participants to enter their year of birth and their age, respectively. We then calculated their age in years based on the year they completed the survey. Regarding cognitive abilities of the elderly, we compare the calculated age and the age they provide. If they were the same or 1 year difference, and they were older than 55 years old, we would keep the sample. Otherwise, we rejected this sample.

##### Gender

We asked participants to enter their gender and provided them with 2 response options: male; female. In the present study, “male” was coded as 1 and “female” was coded as 0.

##### Education level

We asked participants to enter their education level with 5 response options: 1 (primary degrees or below); 2 (secondary degrees); 3 (high school degrees); 4 (associate degrees); 5 (bachelor’s degrees or above). Therefore, higher scores represent higher education level.

##### Number of children

We asked the following question to know number of children of participants:” What is the number of children you have?” and the participants needed to fill in their own answers above the horizontal lines.

##### Living with children

We assessed participants’ living situation with one survey item (Meshi et al., 2020). We asked, “What is your living situation?” and provided participants with the following 4 response options: Alone; Living with spouse; Living with children; Living with other people. With regards to our research topics, in our analyses, “Living with children” was coded as 1 and the rest was coded as 0.

##### Taking care of grandchildren

We asked the following question to know whether the participants need to take care of grandchildren: “Do you currently help your children with childcare?” and provide two response options: yes; no. In the present study, “yes” was coded as 1 and “no” was coded as 0.

##### Estimated daily time on internet

We asked participants the following question to estimate daily time they spend on internet: “How many hours a day do you spend online or on your cell phone?” Then, we provided horizontal lines for participants to fill in their own answers.

## Results

### Preliminary statistics

Harman’s single factor test was conducted to check for the potential effects of common method bias. All scale items were subjected to exploratory factor analysis in SPSS to test for common method variance. The results showed that in the unrotated factor structure, the eigenvalue of the first common factor was 12.40 and the variance explained was 31.00%, which did not reach 50% (Jia et al., 2019), so there was no serious common method bias in the sample data.

In addition, we used confirmatory factor analysis to test for common method bias in MPLUS software. We analyzed all scale measures inside a factor, and the results showed a poor model fit (RMSEA = 0.116; CFI = 0.555, TLI = 0.531; SRMR = 0.130), which indicated that all measures should not belong to the same factor (Jia et al., 2020). Thus, common method bias was unlikely to be of great concern in the sample data. In addition, there was no significant difference between our sample and the non-response sample in terms of age, gender, education level, and t-test. Therefore, the effect of non-response bias is very limited (Wang et al., 2022).

### Descriptive statistics

Table 1 shows the results of descriptive statistics and correlations among all variables, including the means, standard deviations, and the correlation coefficients. It is obvious that there were close relationships between the core variables in the study. Interaction with children was negatively correlated with Internet addiction of the elderly (*r* = −0.145, *p* < 0.05) and loneliness (*r* = −0.231, *p* < 0.001). Additionally, loneliness was positively correlated with Internet addiction of the elderly (*r* = 0.209, *p* < 0.01). Moreover, life satisfaction was related inversely to loneliness (*r* = −0.528, *p* < 0.001) and Internet addiction of the elderly (*r* = −0.145, *p* < 0.05), but positively to interaction with children (*r* = 0.139, *p* < 0.05).

### Hypothesis testing

#### The impact of interaction with children on internet addiction of the old

We used hierarchical regression analysis to test Hypotheses 1. As shown in Table 2, interaction with children was negatively associated with Internet addiction of the elderly (*β* = −0.124, *p* < 0.01; Model 4). Hypothesis 1 was supported.

**Table 2:** Results of hierarchical regression analysis.

Variables	Loneliness				Internet addiction			
	Model 1	Model 2	Model 3	Model 4	Model 5	Model 6	Model 7	Model 8
Control variables								
Age	−0.009	−0.006	−0.007	−0.007	−0.005	−0.007	−0.005	−0.005
Gender	0.013	−0.019	−0.035	−0.072	−0.075	−0.076	−0.072	−0.070
Education level	−0.046	−0.027	−0.017	0.007	0.015	0.009	0.015	0.014
Number of children	0.039	0.042	0.040	−0.056	−0.063^*^	−0.056	−0.064^*^	−0.064^*^
Living with children	0.111	0.008	−0.005	−0.020	−0.040	−0.032	−0.033	−0.032
Taking care of grandchildren	0.159	0.166^*^	0.172^*^	0.276^**^	0.248^**^	0.277^**^	0.244^**^	0.243^**^
Estimated daily time on Internet	0.005	−0.013	−0.009	0.210^***^	0.209^***^	0.208^***^	0.210^***^	0.210^***^
Independent variable								
Interaction with children	−0.168^***^	−0.118^**^	−0.111^**^	−0.124^**^	−0.095^*^	−0.119^**^	−0.096^*^	−0.096^*^
Moderator								
Life satisfaction		−0.412^***^	−0.421^***^			−0.046	0.034	0.037
Interaction 1								
Interaction with children^*^ Life satisfaction			0.120^**^					−0.015^*^
Mediator								
Loneliness					0.174^**^		0.195^**^	0.199^**^
_cons	2.597^***^	2.435^***^	2.439^***^	2.556^***^	1.989^***^	2.676^***^	1.830^***^	1.559^***^
*R^2^*	0.104	0.336	0.356	0.390	0.410	0.392	0.411	0.411

* *p* < 0.05;

** *p* < 0.01, and

*** *p* < 0.001.

*N* = 241.

#### Loneliness as a mediator

To test the mediation hypothesis of the proposed model, we firstly used hierarchical regression analysis to test Hypotheses 2. First, as shown in Table 2, the regression results of Model 1 indicted that interaction with children was negative with loneliness (*β* = −0.168, *p* < 0.001; Model 1), as the control variables were entered into Model 1. Second, based on Model 1, we added the mediator variable to Model 5. And the results represented that interaction with children was negatively correlated with Internet addiction of the elderly, as loneliness was controlled (*β* = −0.095, *p* < 0.05; Model 5). Meanwhile, when interaction with children was controlled, loneliness was positively related to Internet addiction of the elderly (*β* = 0.174, *p* < 0.01; Model 5). Therefore, according to the causal steps approach used by Baron and Kenny (1986), we proposed that loneliness played a mediating role in the relationship between interaction with children and Internet addiction of the elderly, supporting Hypothesis 2.

Secondly, we used bootstrap method to further examine the significance of indirect effect. The results of the mediating effect of loneliness between interaction with children and Internet addiction of the elderly are presented in Table 3, indicating that loneliness partially mediated the association between interaction with children and Internet addiction of the elderly. As predicted, interaction with children appeared to have a negative indirect effect on Internet addiction of the elderly through loneliness. Bootstrap results verified the assumption with a bootstrapped 95% confidence intervals around the indirect effect did not contain zero (−0.086, −0.008). Hence, these findings provided initial support for Hypothesis 2.

**Table 3:** Non-standardized mediation analysis results.

Model paths	Estimate	SE	BC 95% CI	
			Lower	Upper
Total effect				
Interaction with children → Internet addiction	−0.124	0.039	−0.202	−0.047
Direct effect				
Interaction with children → Loneliness	−0.168	0.040	−0.248	−0.088
Loneliness → Internet addiction	0.174	0.063	0.050	0.297
Interaction with children → Internet addiction	−0.095	0.040	−0.175	−0.016
Indirect effect				
Interaction with children → Loneliness → Internet addiction	−0.029	0.014	−0.086	−0.008

BC, biased corrected (5,000 bootstrapping sample). Control variables (age, gender, education level, number of children, living with children, and taking care of grandchildren) were added to the non-standardized mediation analysis.

#### Life satisfaction as a moderator

Life satisfaction was examined as a potential moderator of the association between interaction with children and loneliness. We applied the moderated causal step approach of analysis to examine Hypothesis 3. Based on the data results we provided, there was a significant positive relationship between interaction with children and life satisfaction. As Table 2 indicates, interaction one term was significantly and positively related to loneliness (*β* = 0.120, *p* < 0.01; Model 3). That meant as interaction with children and life satisfaction interacted, life satisfaction enhanced individuals to feel less feelings of loneliness. Hypothesis 3 was supported.

**Figure 2:**
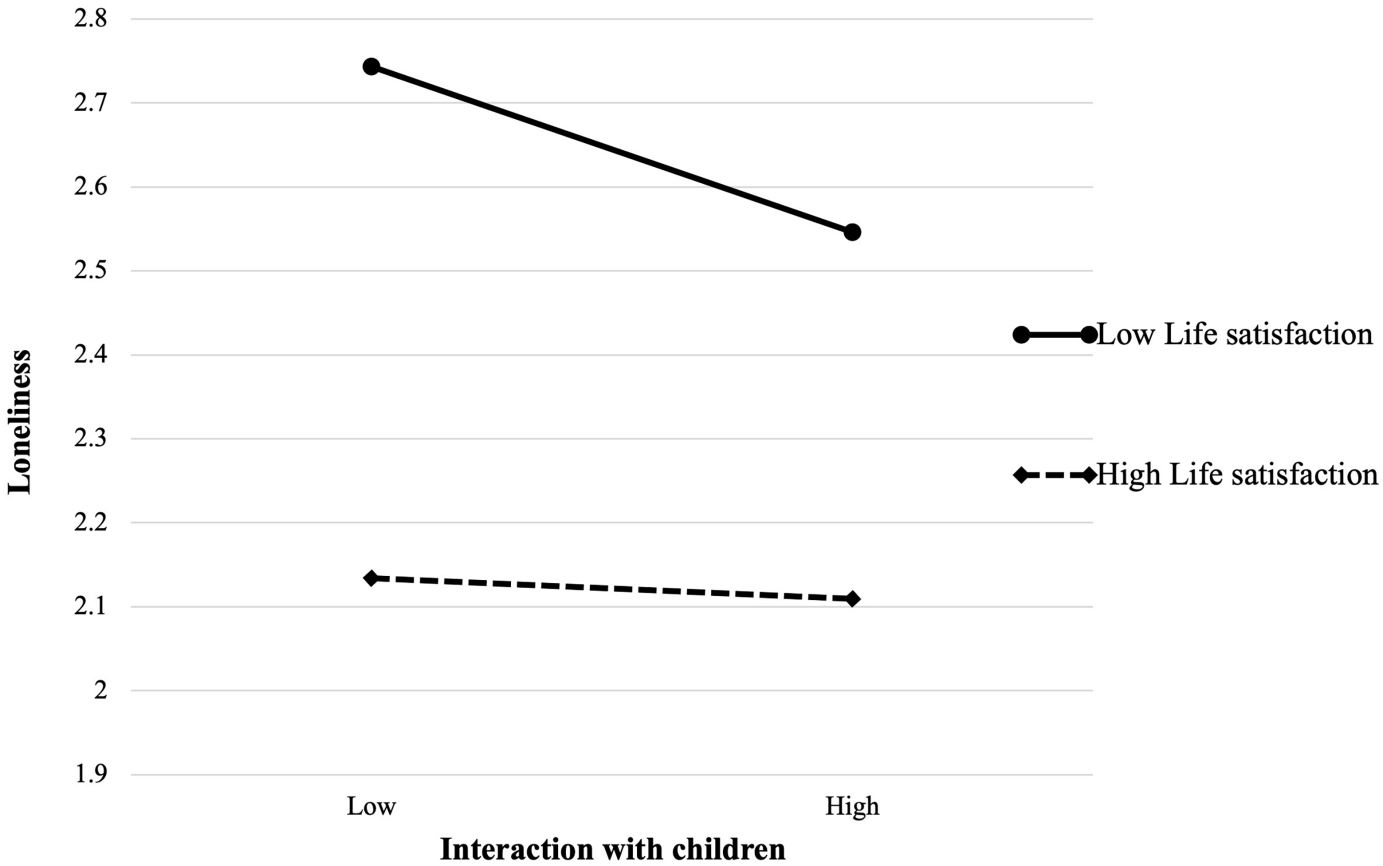
Moderating effect of life satisfaction.

Following the procedure recommended by Stone and Hollenbeck (1989), we further computed the slopes using one SD above (high level of life satisfaction) and below (low level of life satisfaction) the mean of the moderating variable and then plotted the moderation patterns. The potential enhancive effect of interaction with children on loneliness, depicted visually in Figure 2, was interpreted to mean that the negative effect of interaction with children on loneliness would be stronger for older adults with low high life satisfaction than for those with high life satisfaction.

#### Testing for a moderated mediation model

The proposed moderator variable was integrated into the simple mediation model and empirically examined the overall moderated mediation, which was tested by PROCESS macro for SPSS 23.0 with 5,000 bootstrap samples. The data results are displayed in Table 4. As hypothesized, the indirect effect of interaction with children on Internet addiction of the elderly through the psychological mechanism of loneliness was moderated by life satisfaction when the level of life satisfaction was low. In general, the index of moderated mediation indicated that the indirect effect of interaction with children on Internet addiction of the elderly through loneliness depended on life satisfaction. Because the confidence interval (95% CI = 0.002–0.046) did not include zero, with the upper bound positive. The conclusion indicated that the indirect effect of interaction with children on Internet addiction of the elderly through loneliness was negatively moderated by life satisfaction. Specifically, when the elderly possessed low level of life satisfaction, the indirect effect was enhanced (low = −0.034, CI did not include zero). By combining these analyses, Hypothesis 4 was well demonstrated.

**Table 4:** Moderated mediation results.

Moderator variable	Estimate	SE	BC 95% CI	
			Lower	Upper
Life satisfaction low (-1SD)	−0.034	0.016	−0.068	−0.007
Life satisfaction high (+1SD)	−0.004	0.010	−0.026	0.014
Index	0.021	0.012	0.002	0.046

BC, biased corrected (5,000 bootstrapping sample). Control variables (age, gender, education level, number of children, living with children, and taking care of grandchildren) were added to the non-standardized mediation analysis.

## Discussion

The present study’s goal was to explore the relationship between interaction with children and Internet addiction of the older adults, as well as the mechanisms of the relationship. More and more older adults get used to the Internet use to meet their emotional and informational needs. So, more attention should be paid to the older adults’ Internet addiction, as the old are prone to use the internet excessively because of their lack of social support and physical activity and experience high loneliness when they are addicted to the internet (Meshi et al., 2020). Although existing research findings have showed that social support and loneliness are strong predictors of Internet addiction (Bozoglan et al., 2013; Wu et al., 2016; Özbek and Karaş, 2022), it is an intriguing question whether the emotional support of family (e.g., interaction with children) has a unique effect on Internet addiction among the old, or whether this association will be mediate by loneliness. Besides, results from the role of life satisfaction in the prevention of loneliness has revealed that life satisfaction is devoted to reducing feelings of loneliness (Andrew and Meeks, 2018) and life satisfaction can help explain why some are more prone to Internet addiction while others are less (Lachmann et al., 2018). It is worth thinking whether the indirect effect linking interaction with children and Internet addiction of the older adults through loneliness will be moderated by life satisfaction. Therefore, the current study examined whether loneliness mediated the relation between interaction with children and Internet addiction of the older adults, and whether this indirect effect was moderated by life satisfaction.

Four important hypotheses needed to be investigated. The first hypothesis was to examine the impact of interaction with children on the Internet addiction among the old. The results of present study revealed that interaction with children has a negative effect on the elderly’s Internet addiction. The study’s findings were consistent with earlier research. Prior literature supports the hypothesis of this research that interaction with children can reduce the elderly’s Internet addiction. As previous literature has examined that interpersonal relationships and social support are significantly related to Internet addiction (Yeh et al., 2008; Tian et al., 2017) and better intergenerational relationships between older adults and their children mean more family support for Chinese older adults (Li et al., 2019). In addition, some studies have confirmed that intergenerational relationships with children affect older adults’ Internet use (Yuan et al., 2022) and social support from family and from a significant other can reduce the older adults’ addictive use of social media (Özbek and Karaş, 2022). Taken together, interactions with children can reduce Internet addiction in older adults while improving their skill to internet use.

The second hypothesis was to find the role of loneliness as a moderator, the association between interaction with children and the Internet addiction among the old. The results of this study revealed that increased interaction with children would reduce the level of loneliness, so that Internet addiction of the old would decrease. The study’s findings were consistent with earlier research. Prior literature supports the hypothesis of this research that loneliness plays a mediating role on the relationship between interaction with children and the Internet addiction among the old. As previous studies suggested that loneliness was a mediator on the association between quality of social relationships and Internet addiction among college students (Guo et al., 2020). Additionally, the relationship between interpersonal relationships and loneliness, and the relationship between loneliness and Internet addiction has been clearly demonstrated (Błachnio et al., 2016; Tian et al., 2019). These findings indicate that increased interaction with children would reduce the level of loneliness, so that Internet addiction of the old would decrease. In conclusion, the alleviation of loneliness is preventative for older people, which can prevent from Internet addiction.

The third hypothesis was to find the role of life satisfaction as a moderator, the association between interaction with children and loneliness. And the fourth hypothesis was to test whether life satisfaction moderates the indirect effects of interaction with children on Internet addiction among the old through loneliness. The results of present research suggested that compared to those older adults who have high life satisfaction, those who have low life satisfaction would experience an enhancive impact with the negative effect of interaction with children on loneliness. Besides, life satisfaction moderates the indirect effects of interaction with children on older adults’ internet addiction through loneliness. The study’s findings were consistent with earlier research and prior literature supports the hypothesis of this research. As previous studies have indicated that life satisfaction is a potentially important protective factor against adverse or stressful life events (Chai et al., 2018). Moreover, life satisfaction is considered to be a general evaluation style that will influence people’s immediate cognitive evaluations of specific environmental events (Lazarus, 1993). These findings underscored that, people with lower life satisfaction will develop coping strategies in a more negative manner, which can enhance the positive influence of advantageous resources on individuals.

### Implications of theory

The current study constructed and examined a model that distinguished the factors which aggravate or alleviate older adults’ Internet addiction. Previous literature focused more exclusively on the internet addiction in young and middle-adult groups and the benefits of the Internet usage of the elderly. However, few studies, to our knowledge, noticed the impact of family factors on Internet addiction in older adults, as well as the mechanisms of the impact. To fill in these gaps, we conducted an empirical analysis about these topics among Chinese older adults. Consequently, the results of the present study have theoretical significance.

First, according to the social support theory, the present study suggested the negative impact of interaction with children on Internet addiction of the old. Previous studies have suggested that social support is an of vital variable to reducing Internet addiction (Wang and Zhang, 2020) and interpersonal relationships are significantly related to Internet addiction (Tian et al., 2017). But these studies are conducted among youth and mid-age group. Additionally, family is such an important place where older adults can feel social connection. Better intergenerational relationships between older adults and their children mean more family support. Therefore, it is worth exploring whether interaction with children can prevent or reduce Internet addiction in older adults. Thus, the findings of this research extended the study boundary of the Internet addiction area, explored a mechanism to mitigate Internet addiction in older adults and enriched theories of family intergenerational relations.

Second, the results of our study supported the psychosocial distress model and demonstrated an important mediating mechanism of how interaction with children effect the elderly’s Internet addiction. In other words, loneliness played a mediating role on the relationship between interaction with children and Internet addiction of the old. Older adults need to cope with the challenges of decreased social support and increased loneliness, which leads them to choose to supplement these needs through internet use (Liu and Ma, 2019). However, long-term Internet use also comes with new life challenges, namely Internet addiction. To effectively reduce or prevent Internet addiction, it is worth exploring the mechanism of the negative effect of interaction with children on Internet addiction for Chinese older adults who place more emphasis on family life. Therefore, this study extends the research on the mitigating mechanisms of Internet addiction in older adults and verifies the mediating role of loneliness.

Thirds, the present analysis indicated that life satisfaction moderated the relationship between interaction with children and Internet addiction through loneliness. High life satisfaction can influence personal health by enhancing psychological and social resources (Kim et al., 2017). However, because of the loss of more social interactions, older adults often face situations where social support decreases and life satisfaction decreases (Shillair et al., 2015). It has been established that high social support is associated with high life satisfaction and high life satisfaction is associated with low internet addiction (Momeni and Rafiee, 2018). On the other hand, life satisfaction plays a protective role for individuals from loneliness (Andrew and Meeks, 2018). Then, the role that life satisfaction plays in the mechanism of Internet addiction mitigation in older adults is worth exploring. Therefore, this paper extends the research on the role of life satisfaction in older adults and further explores its role in the mechanism of Internet addiction mitigation in older adults.

### Implications of practice

In additional, the results of the present study have practical implications. Researchers who conducted the internet use of the old should notice our findings and pay attention to the Internet addiction of the old individuals. Therefore, this study proposes three specific methods to reducing or prevent the old people’s Internet addiction.

The first measure is to strengthen the family support system of older adults. Older adults who maintain close ties with their families have better mental health than socially isolated older adults (Wang et al., 2020). This is because older adults feel loved and valued during interactions with their families, which can reduce their feelings of isolation and risk of developing illnesses. Improved family support systems and elevated child support can alleviate some of the life stresses that can weaken the health of older adults. Therefore, families need to play a greater role in reducing loneliness and Internet addiction in older adults. When developing the interventions to the elderly’s loneliness and Internet addiction, improving the interaction with children can help prevent the development of Internet addiction in these people, especially for older people with low life satisfaction.

The second measure is for the community to make good use of the elderly’s free time to enhance their social connection and social participation. Social participation has a suppressive effect on the loneliness of the elderly (Zhao and Wu, 2022). In other words, the elderly can strengthen their social connection with other people in social participation, from which they can get social support and sense of belonging, which can reduce the loneliness. And after the offline needs of interpersonal interaction, social support and sense of belonging are fulfilled, individuals will not indulge with satisfying these needs from the Internet (Milani et al., 2009). Therefore, communities can enrich the social and recreational life of the elderly, which can both exercise and gradually get rid of Internet addiction.

The third measure is to guide older adults to use the Internet properly to enhance their life satisfaction. Healthy Internet use is extremely beneficial to older adults and can enhance their life satisfaction (Lam et al., 2020). Life satisfaction can influence individual health and behavior by increasing psychological and social resources (Kim et al., 2017). Therefore, the state should make age-appropriate modifications at the levels of technology, products, and services to gradually bridge the digital divide while guiding the elderly to use the Internet properly. Additionally, children should also shoulder the responsibility of monitoring, guiding their parents to surf the Internet moderately and teaching them to recognize online information.

### Limitations and future directions

Although our research has theoretical and practical implications, there are some limitations. First, the relevant measurements in the paper were based on the results of self-reported data, with there was a risk of obscuring the objective factual picture. Therefore, future studies need more objective measurements to verify the stability and reliability of research findings in this paper. Second, this paper only paid attention to older adults with children, but in fact there are some older adults without children who may be more at risk of Internet addiction. Therefore, the generalizability of our study to older adults without children is limited. However, considering the Chinese clan consciousness and the reality that most Chinese older adults have children and that intergenerational relationships are important throughout the Chinese life cycle (Li and Dong, 2020), our study is still valuable. Consequently, for those older adults who may lack a source of support from the family system in their later years, it is of greater concern how to enhance their other social support systems. Third, this paper did not focus on the mechanisms of personality effects on the relationship between interaction with children, loneliness, and Internet addiction. It has been established that Internet addiction is significantly associated with individual personality traits (Karaoglan Yilmaz et al., 2022). Thus, considering the effects of personality would help us improve interventions for Internet addiction in older adults in future research.

## Conclusion

In summary, our findings reveal the value of interactions with children in older adults with Internet addiction. We found that interactions with children can reduce Internet addiction in older adults while protecting their psychological well-being, which means reduced feelings of isolation. More importantly, for those older adults with low life satisfaction, interactions with children are more likely to reduce loneliness and thus Internet addiction. Researchers should pay attention to this relationship between interactions with children, loneliness, life satisfaction, and Internet addiction in older adults. In addition, future research could build on our current findings to develop potential interventions for Internet-using older clients to mitigate or prevent their Internet addiction, with the aim of increasing real-life family support.

## Data availability statement

The raw data supporting the conclusions of this article will be made available by the authors, without undue reservation.

## Ethics statement

The studies involving human participants were reviewed and approved by School of Sociology, Wuhan University. Written informed consent for participation was not required for this study in accordance with the national legislation and the institutional requirements

## Author contributions

YY contributed to writing original draft, conceptualization, data curation, formal analysis, and methodology. TL contributed to resources, data collection, and supervision of the paper. YJ contributed to data curation, methodology, review, and editing. All authors contributed to the article and approved the submitted version.

## Funding

This work is supported by the National Natural Science Foundation of China [72102170; 72172107] and Independent Research Project of Wuhan University [2021XWZY009].

## Conflict of interest

The authors declare that the research was conducted in the absence of any commercial or financial relationships that could be construed as a potential conflict of interest.

## Publisher’s note

All claims expressed in this article are solely those of the authors and do not necessarily represent those of their affiliated organizations, or those of the publisher, the editors and the reviewers. Any product that may be evaluated in this article, or claim that may be made by its manufacturer, is not guaranteed or endorsed by the publisher.
